# The Effects of Hemodialysis on Tear Osmolarity

**DOI:** 10.1155/2015/170361

**Published:** 2015-11-10

**Authors:** Muhittin Taskapili, Kubra Serefoglu Cabuk, Rukiye Aydin, Kursat Atalay, Ahmet Kirgiz, Dede Sit, Hasan Kayabasi

**Affiliations:** ^1^Prof. Dr. N. Resat Belger Beyoglu Eye Training and Research Hospital, 34421 Istanbul, Turkey; ^2^Ophthalmology Department, Bagcilar Training and Research Hospital, 34200 Istanbul, Turkey; ^3^Ophthalmology Department, Medipol University, 34214 Istanbul, Turkey; ^4^Nephrology Department, Bagcilar Training and Research Hospital, 34200 Istanbul, Turkey

## Abstract

*Aim*. To determine the effects of hemodialysis (HD) on tear osmolarity and to define the blood biochemical tests correlating with tear osmolarity among patients with end stage renal disease (ESRD).* Material-Method*. Tear osmolarity of ESRD patients before and after the hemodialysis program was determined as well as the blood biochemical data including glucose, sodium, potassium, calcium, urea, and creatinine levels.* Results*. Totally 43 eyes of 43 patients (20 females and 23 males) with a mean age of 53.98 ± 18.06 years were included in the study. Tear osmolarity of patients was statistically significantly decreased after hemodialysis (314.06 ± 17.77 versus 301.88 ± 15.22 mOsm/L, *p* = 0.0001). In correlation analysis, pre-HD tear osmolarity was negatively correlated with pre-HD blood creatinine level (*r* = −0.366,  *p* = 0.016). Post-HD tear osmolarity was statistically significantly correlated with the post-HD glucose levels (*r* = 0.305  *p* = 0.047). Tear osmolarity alteration by HD was negatively correlated with creatinine alteration, body weight alteration, and ultrafiltration (*r* = −0.426,  *p* = 0.004; *r* = −0.365,  *p* = 0.016; and *r* = −0.320, *p* = 0.036, resp.). There was no correlation between tear osmolarity and Kt/V and URR values.* Conclusion*. HD effectively decreases tear osmolarity to normal values and corrects the volume and composition of the ocular fluid transiently. Tear osmolarity alteration induced by HD is correlated with body weight changes, creatinine alterations, and ultrafiltration.

## 1. Introduction

Hemodialysis (HD) is the main treatment method in patients with end stage renal disease (ESRD) to correct the composition and volume of body fluids. The adequacy of HD is still a main subject for active investigation [1, 2].

There are some parameters present for evaluation of adequacy of HD such as *Kt*/*V* (a number used to quantify hemodialysis treatment adequacy, in which *K* is the dialyzer clearance of urea, *t* is the dialysis time, and *V* is the volume of distribution of urea) and urea reduction ratio (URR) [3].

HD may alter the volume and composition of ocular fluid as well as the systemic hemodynamic parameters [4]. Presence of dry eye in patients with ESRD is known for years [5–7].

Dry eye disease is an important and common public health problem with 5–35% prevalence in general population, as it causes discomfort and deterioration in quality of vision [8]. Although, a direct method is still not present, dry eye questionnaires, Schirmer's test, and tear break-up time (TBUT) are in clinical use to support the dry eye diagnosis. Nowadays, tear osmolarity measurement is regarded as the most accurate way of diagnosis of dry eye disease [9]. As previous studies showed, reduction in aqueous tear flow as a result of lacrimal failure with or without accelerated evaporation from the tear film is major determinants of the tear hyperosmolarity [8, 10]. Additionally, the electrolytes of the aqueous phase of the tear film can effect tear osmolarity [11]. Of the electrolytes present in the tear film, cations sodium and potassium (120–170 mmol/kg and 6–42 mmol/kg, resp.) and anions chloride and bicarbonate (106–135 mmol/kg and 26 mmol/kg, resp.) are the major contributors to tear osmolarity [12]. Measurement of tear osmolarity in clinical setting is easy with recently developed lab-on-a-chip technology, namely, TearLab (TearLab Corporation, San Diego, CA, USA), with 72.8% sensitivity and 92.0% specificity at a cutoff value of 312 mOsms/L [9].

In this study, we aimed to determine the effects of HD, performed with isovolemic and standard sodium (Na^+^) (138 mEq/L) and potassium (K^+^) (2 Eq/L) containing dialysates, on tear osmolarity and to evaluate the correlation between blood biochemical tests and tear osmolarity in patients with ESRD.

## 2. Material and Method

### 2.1. Patients

Tear osmolarity of 43 eyes of 43 patients under the regular, 3 times per week, hemodialysis program in Bagcilar Education and Research Hospital, Hemodialysis Unit, was evaluated and McMonnies and Ho questionnaire was filled in between April 2014 and June 2014. The blood samples were taken and tear osmolarity was detected one minute before the beginning of the hemodialysis and 30 minutes after the termination of hemodialysis program. Patients with diabetic retinopathy and any rheumatic and connective tissue diseases, patients using any type of eye drops and wearing contact lenses, and patients with the history of ocular surgery were excluded from the study.

The study protocol was approved by the local ethics committee in Bagcilar Education and Training Hospital, Turkey. Informed consent was obtained from all subjects.

### 2.2. Laboratory Tests

Pre-HD blood samples were taken and tear osmolarity was detected one minute before the beginning of HD. The rate of diffusion and blood flow between body compartments reduce the effective *K* and therefore *Kt*/*V* and result in the postdialysis rebound. To take account of these factors, *Kt*/*V* should ideally be calculated using a postdialysis sample taken 30–60 minutes after dialysis when the urea concentrations have reequilibrated [13]. As urea enters into tear fluid by simple diffusion [14], post-HD tear osmolarity was detected and biochemical samples were taken 30 minutes after the termination of HD session. Body weights of participants were recorded as well as the ultrafiltration amount. Serum urea, creatinine, glucose, sodium, potassium, calcium, and bicarbonate (HCO_3_^−^) levels were studied using the standard methods recommended by the manufacturer. Serum osmolarity was calculated with the formula [15]:

(1) Serum osmolarity=2Na++Glucose18+Blood urea nitrogen2.8.

The urea reduction ratio (URR) is a number used to quantify dialysis treatment adequacy and similarly *Kt*/*V* is also a number used to quantify hemodialysis treatment adequacy, in which *K* is the dialyzer clearance of urea, *t* is the dialysis time, and *V* is the volume of distribution of urea.

URR was calculated as a percentage of post-HD blood urea nitrogen (BUN) divided by pre-HD BUN. The single-pool *Kt*/*V* delivered by hemodialysis was estimated by the second-generation Daugirdas equation [16].

We have recorded the URR and *Kt*/*V* and investigated their associations with tear osmolarity.

Dry eye symptoms of the patients are evaluated with McMonnies and Ho questionnaire. Any score over 14.5 indicates a strong likelihood of dry eye disease [17]. Tear osmolarity was measured using lab-on-a-chip technology TearLab Osmolarity System (TearLab Corporation, 9980 Huennekens Street, Ste 100, San Diego, CA 92121, 1-855-832-7522, USA), one minute before the beginning of HD and 30 minutes after the termination of HD. The measurements were performed at a stable room temperature of 25–25.5°C and the room humidity was 50–55%. Quality control procedures were applied at the beginning of each day of patient testing by using reusable electronic check cards (provided by the manufacturer as a procedural quality control) to confirm the function and calibration of the TearLab Osmolarity System. A tear sample, approximately 50 nL, was collected from the inferior lateral tear meniscus of the ocular surface by the same investigator.

### 2.3. Statistical Analysis

Statistical analysis was performed using Statistical Package for Social Sciences (SPSS, SPSS Inc., Chicago, IL, USA) version 21.0. Descriptive statistics were summarized as mean ± SD or percentage. Paired samples *t*-test was performed in comparison of pre-HD and post-HD results. Chi square test was used in comparison of two groups. Pearson's correlation analysis was performed to determine the correlations of laboratory data with tear osmolarity. A *p* value of <0.05 was regarded as statistically significant.

## 3. Results

Totally, 43 eyes of 43 patients (20 females and 23 males) with a mean age of 53.98 ± 18.06 years were included in the study.

McMonnies and Ho questionnaire was performed before HD to 41 patients because 2 patients were unable to complete the questionnaire. Mean questionnaire score was 6.27 ± 5.02. And all patients gave the same answers to the questionnaire after HD. There was only one patient who had dry eye according to the McMonnies and Ho questionnaire.

Descriptive characteristics of study participants are summarized in Table 1.

In the 37 of the 43 patients (86%) URR was over 65% and *Kt*/*V* was over 1.2 and HD was adequate according to these two parameters.

Body weight, serum urea, creatinine, calcium, and potassium, and tear osmolarity of patients statistically decreased, and HCO_3_^−^ increased significantly after HD (*p* = 0.0001) (Table 2).

There was no significant correlation between pre-HD tear osmolarity and pre-HD serum osmolarity, glucose, urea, sodium, potassium, calcium, and bicarbonate levels and body weight (*p* > 0.05). Pre-HD tear osmolarity was statistically significantly correlated with pre-HD creatinine (*r* = −0.366  *p* = 0.016).

There was no significant correlation between post-HD tear osmolarity and post-HD serum urea, creatinine, sodium, potassium, calcium, and bicarbonate levels and body weight (*p* > 0.05). Post-HD tear osmolarity was statistically significantly correlated with post-HD glucose (*r* = 0.305, *p* = 0.047).

We also subgrouped patients according to the presence of diabetes mellitus type 2 (DM) and hypertension (HT). The difference regarding pre-HD and post-HD tear osmolarity between patients with or without DM was not statistically significant (*p* > 0.05) (Table 3).

The difference regarding pre-HD tear osmolarity between patients with or without HT was not statistically significant (*p* > 0.05). But post-HD tear osmolarity of patients without HT was statistically significantly lower than patients with HT (*p* = 0.043) (Table 4).

In correlation analysis, tear osmolarity difference was statistically significantly correlated with ultrafiltration, body weight difference, and creatinine difference but not with URR and *Kt*/*V* values. The *p* values of correlation analysis are summarized in Table 5.

## 4. Discussion

The adequacy of HD is important for management of patients with ESRD. According to latest guidelines the minimally adequate dose of HD given 3 times per week to patients with *K*_*r*_ less than 2 mL/min/1.73 m^2^ should be sp*Kt*/*V* of 1.2 per dialysis. For treatment less than 5 hours, an alternative minimum dose is URR of 65% [3]. In our study HD achieved minimally adequate doses in 37 patients (86%) but not in 6 patients.

In an adequate hemodialysis serum sodium levels are determined as 135–145 mEq/L, potassium 3–9 mEq/L, calcium 7–12 mEq/L, bicarbonate > 15 mEq/L, creatinine < 12 mEq/L, and albumin >3 gr/dL [3]. In our study all of these biochemical markers were in these ranges.

In this study, we have evaluated tear osmolarity of patients with ESRD one minute before the beginning of HD and 30 minutes after the end of HD. We observed tear hyperosmolarity before HD and a significant reduction to normal levels after HD (314.05 ± 17.77 mOsm/L and 301.88 ± 15.22 mOsm/L, resp., *p* < 0.0001). Gilbard et al. indicate in two rabbit models for keratoconjunctivitis sicca that decreased tear volume or excessive evaporation is the major cause of tear hyperosmolarity [10]. Charlton et al. report tear hyperosmolarity, using freezing point depression method, (average 347 mOsm/L, range 375–312 mOsm/L) in 10 renal dialysis patients in pre-HD and tested 5 of them immediately after completion of HD. They show a significant reduction in tear osmolarity after HD in all patients, correlating with our results. They speculate that, from the three principle solutes (sodium, glucose, and urea), urea is the only one that freely passes from serum to the tears and responsible for tear hyperosmolarity in renal dialysis patients [18].

In our study only one patient scored positively for dry eye before HD according to the McMonnies and Ho questionnaire and all patients gave the same answers to the questionnaire after HD. Similarly, Charlton et al. report that none of the renal dialysis subjects scored positively for dry eye. They speculate that, from the three principle solutes (sodium, glucose, and urea), urea is the only one that freely passes from serum to the tears and is responsible for tear hyperosmolarity in renal dialysis patients. According to them, hemodialysis patients remain asymptomatic for dry eye mainly because of the protective effects of urea in tears on the ocular surface [18].

In this study, we did not find any correlation between serum electrolyte levels and tear osmolarity both in pre-HD and post-HD periods. Aktaş et al. determined a prognostic importance of serum calcium levels for the ocular findings and symptoms in patients with ESRD [5]; however, we did not determine any correlation between pre-HD and post-HD calcium levels and tear osmolarity. Serum osmolarity also did not correlate with tear osmolarity.

There was no significant correlation between pre-HD tear osmolarity and pre-HD glucose and urea levels and body weight (*p* > 0.05). Pre-HD tear osmolarity was statistically significantly correlated with pre-HD creatinine (*r* = −0.366, *p* = 0.016). This is the first report denoting an association between tear osmolarity and creatinine according to our knowledge.

There was no significant correlation between post-HD tear osmolarity and post-HD serum urea and creatinine levels and body weight (*p* > 0.05). Post-HD tear osmolarity was statistically significantly correlated with post-HD glucose (*r* = 0.305  *p* = 0.047).

In correlation analysis tear osmolarity difference was statistically significantly correlated with ultrafiltration (*r* = −0.320, *p* = 0.036), body weight difference (*r* = −0.365, *p* = 0.016), and creatinine difference (*r* = −0.426, *p* = 0.004).

In another study among patients undergoing HD for ESRD, the incidence of reduced basal tear secretion and dry eye symptoms were reported to be higher in diabetic patients than in nondiabetics [19]. However, in our study, we have subgrouped patients according to the presence of DM and we did not determine any significant effects of diabetes in pre-HD or post-HD tear osmolarity. Post-HD tear osmolarity of patients with HT was statistically significantly higher than patients without HT (*p* = 0.043). Studies with larger sample sizes are needed for exact relevance of these results.

Jung et al. reported decrease in tear break-up time (TBUT) and Schirmer's tests after HD [20]. We did not evaluated Schirmer's test and TBUT because of low patient compliance after HD.

This study has some limitations. As HD is a long lasting treatment method, patients may be divided into subgroups according to duration of HD. We have evaluated patients regardless of the duration of HD. Lack of Schirmer's test and TBUT are two other limitations. We could not do Schirmer's test and TBUT because of low patient compliance after HD. *Kt*/*V* was <1.2 and URR was <65% and HD was not adequate in only 6 patients. More patients are needed to assess the association between tear osmolarity and adequacy of HD.

In conclusion tear osmolarity is correlated with the serum creatinine levels in pre-HD period. In post-HD period, tear osmolarity is correlated with serum glucose levels. Tear osmolarity alteration induced by HD is correlated with body weight changes, creatinine alterations, and ultrafiltration. Therefore, HD corrects the volume and composition of the ocular fluid transiently.

## Figures and Tables

**Table 1:** Clinical characteristics of the patients.

Gender (F/M)	20/23
Age (years)	53.98 ± 18,06
Time of dialysis (years)	5.12 ± 5.67
Presence of hypertension	32 (76%)
Presence of diabetes mellitus	16 (38%)
Mean URR (%)	73.52 ± 6.1
Mean sp*Kt*/*V*	1.42 ± 0.16

**Table 2:** The laboratory data analysis of patients before and after HD.

	Before HD	After HD	*p*
Body weight	70.59 ± 15.95	68.54 ± 15.57	**0.0001**
Tear osmolarity (mOsm/L)	314.05 ± 17.77	301.88 ± 15.22	**0.0001**
Serum glucose (mg/dL)	128.63 ± 92.26	121.77 ± 42.66	0.533
Serum urea (mg/dL)	130.96 ± 31.03	34.88 ± 12.4	**0.0001**
Serum creatinine (mg/dL)	8.08 ± 2.36	2.87 ± 1.01	**0.0001**
Serum calcium (mEq/L)	8.79 ± 1,16	9.43 ± 0.71	**0.0001**
Serum sodium (mEq/L)	137.28 ± 3.73	136.47 ± 2.69	0.165
Serum potassium (mEq/L)	5.22 ± 0.79	3.96 ± 0.66	**0.0001**
Serum HCO_3_^−^ (mEq/L)	20.59 ± 1.8	23.1 ± 3.5	**0.0001**

The data are reported in mean ± standard deviation. Results of paired samples *t*-test.

**Table 3:** Effects of DM on pre-HD and post-HD tear osmolarity.

	DM	*n*	Mean ± SD	*p*
Pre-HD tear osmolarity	DM (−)	11	319.18 ± 18.8	0.655
	DM (+)	7	323.43 ± 21.10	

Post-HD tear osmolarity	DM (−)	11	302.10 ± 14.06	0.413
	DM (+)	7	308.86 ± 20.23	

**Table 4:** Effects of HT on pre-HD and post-HD tear osmolarity.

	HT	*n*	Mean ± SD	*p*
Pre-HD tear osmolarity	HT (−)	10	311.40 ± 16.77	0.482
	HT (+)	27	316.00 ± 17.71	

Post-HD tear osmolarity	HT (−)	10	295.50 ± 11.21	**0.043**
	HT (+)	27	306.29 ± 14.66	

**Table 5:** Correlation analysis.

		Tear osmolarity difference
Ultrafiltration	*r*	−0.320
	*p*	**0.036**

Body weight difference	*r*	−0.365
	*p*	**0.016**

Creatinine difference	*r*	−0.426
	*p*	**0.004**

URR	*r*	0.057
	*p*	0.718

*Kt*/*V*	*r*	0.055
	*p*	0.728

The results of Pearson's correlation analysis. *r*: correlation coefficient; *p*: statistical significance.
